# Cyclodextrin Stabilized Freeze-Dried Silica/Chitosan Nanoparticles for Improved Terconazole Ocular Bioavailability

**DOI:** 10.3390/pharmaceutics14030470

**Published:** 2022-02-22

**Authors:** Nada Zaghloul, Nada M. El Hoffy, Azza A. Mahmoud, Nermeen A. Elkasabgy

**Affiliations:** 1 Department of Pharmaceutics and Pharmaceutical Technology, Faculty of Pharmacy, Future University in Egypt, Cairo 11835, Egypt; nada.hussein@fue.edu.eg (N.Z.); nada.mohamed@fue.edu.eg (N.M.E.H.); azza.ahmed@fue.edu.eg (A.A.M.); 2 Department of Pharmaceutics and Industrial Pharmacy, Faculty of Pharmacy, Cairo University, Kasr El-Aini Street, Cairo 11562, Egypt

**Keywords:** terconazole, tetraethyl ortho silicate, chitosan HCl, freeze drying, silica nanoparticles, ocular delivery

## Abstract

This research assesses the beneficial effects of loading terconazole, a poorly water-soluble antifungal drug in silica/chitosan nanoparticles (SCNs) for ocular delivery. Nanoparticles were fabricated by the simple mixing of tetraethyl ortho silicate (TEOS) and chitosan HCl as sources of silica and nitrogen, respectively, along with alcoholic drug solution in different concentrations. Freeze-dried nanoparticles were fabricated using cyclodextrins as cryoprotectants. SCNs were assessed for their particle size, PDI, yield, drug loading and in vitro release studies. A 2^3^.3^1^ full factorial experimental design was constructed to optimize the prepared SCNs. DSC, XRD, FTIR, in addition to morphological scanning were performed on the optimized nanoparticles followed by an investigation of their pharmacokinetic parameters after topical ocular application in male Albino rabbits. The results reveal that increasing the water content in the preparations causes an increase in the yield and size of nanoparticles. On the other hand, increasing the TEOS content in the preparations, caused a decrease in the yield and size of nanoparticles. The optimized formulation possessed excellent mucoadhesive properties with potential safety concerning the investigated rabbit eye tissues. The higher C_max_ and AUC_0–24_ values coupled with a longer t_max_ value compared to the drug suspension in the rabbits’ eyes indicated the potential of SCNs as promising ocular carriers for poorly water-soluble drugs, such as terconazole.

## 1. Introduction

Corneal diseases, second to cataracts, represent a major cause of blindness, where most regions of the eye are vulnerable to fungal infections [1,2,3]. Fungal keratitis is considered as one of these diseases [4]. It is caused by several fungal species capable of colonizing in the ocular tissues, with Candida species representing the most common of them [5]. Although Candida species are responsible for the majority of ocular fungal infections, yet, they are still challenging as they are resistant to the most commonly used antifungal drugs [6,7,8]. Previously, the first line to treat fungal keratitis was through the topical administration of amphotericin B, however patients suffered from poor vision as a result of this. Moreover, in some complicated cases, corneal grafts were required [9]. Alternative approaches for using topical amphotericin B were introduced, namely, the utilization of topical azoles. Fluconazole, voriconazole and terconazole proved to have less toxicity on corneal epitheliums, with better ocular tolerability than the latter [10,11,12].

Terconazole is a triazole potent broad-spectrum antifungal drug that possesses pronounced activity against yeast- and mycelium-forming fungi by inhibiting the cytochrome P-450-dependent 14α-demethylase in the fungal membranes; hence leading to ergosterol depletion [13,14]. However, its poor aqueous solubility limited its use [15]. 

Nanoparticles in general possess vast benefits in the drug delivery field, especially when loaded with antimicrobials or antifungals, offering their advantages through improved drug loading, targeting and less drug dosing due to a better uptake by pathogens [16,17]. Silica nanoparticles represent one of the recently introduced nanoplatforms in the drug delivery field [18,19,20]. They offer several advantages, such as the ease of fabrication, better drug encapsulation, enhanced drug solubility as well as controlling drug release [21]. Moreover, they are considered a biocompatible platform with low toxicity when administered through different routes. The in vivo safety profile of silica nanoparticles has been previously addressed, offering a propitious approach for the ocular topical delivery of drugs [22,23]. Using silica nanoparticles as drug carriers for ocular delivery has been introduced several times in the literature [24,25,26].

DelSiTech^TM^Silica is a technology that develops implant and eye drop dosage forms based on biodegradable silica matrices, where the release of the drug depends on the erosion of the biodegradable silica in the ocular tissue. DelSiTech’s long-acting injectable intravitreal depot formulation for treating retinal inflammation is currently in the preclinical stage. This formulation is able to release an anti-inflammatory compound for up to 3 months [27].

Several methods were applied for the preparation of silica particles, including a cross-linked copolymer, which implements the loading of the drug on mesoporous silica cross-linked with polymers [28]. Unfortunately, most of the applied methods for manufacturing silica particles require harsh conditions with prolonged exposure to elevated temperatures that may reach 800 °C for 24 h [29,30], which in turn limits their use with thermolabile drugs. Furthermore, undesirable irregular shaped nanoparticles were obtained in many cases [31,32]. The microemulsion method succeeded in the preparation of particles with a particle size range between 30 and 60 nm. Nonetheless, large amounts of surfactants exceeding the silica weight are needed to produce inverse micelles, which necessitates post-treatment approaches to remove excess surfactants [33]. Other fabrication techniques include the aqueous hydrolysis of finely-divided elemental silicon, using ammonia as a catalyst in the temperature range of 20–90 °C [34]. Although this is a reasonable temperature range, this method requires the activation of the elemental silicon before the reaction by washing with aqueous hydrofluoric acid, pure water, alcohol and ether, consecutively, to remove silicon dioxide film from the particle surface and to expose a clean silicon surface [35].

Polysaccharides are widely used as ocular drug delivery carriers [36,37]. Chitosan HCl is a mucoadhesive cationic polysaccharide polymer with considerable capability for topical ocular drug delivery [38]. As the cornea and conjunctiva are anionic in nature, the use of cationic polymers would assist molecular attractions through electrostatic interactions, hence increasing the residence time of the formulation and offering more sustained drug delivery [39,40]. Additionally, chitosan is a biocompatible and biodegradable polymer with a proved good safety profile [41,42,43].

For many years, cyclodextrins were recognized as effective pharmaceutical excipients [44]. Their hollow torus-shaped molecules comprising a lipophilic cavity and hydrophilic outer surface enable them to solubilize hydrophobic drugs through an inclusion complexation process [45]. β-cyclodextrin (βCD) has many derivatives, such as hydroxypropyl-β-CD (HβCD) as well as methyl-β-CD (MβCD). Those derivatives are of a higher aqueous solubility compared to the parent βCD [46]. Moreover, both HβCD and MβCD are available as pharmaceutical excipients in marketed eye drops, i.e., Indocollirio (Bausch & Lomb, France) and Clorocil (Oftalder, Poland), respectively. Another added value for the use of cyclodextrins is their ability to protect nanoparticles during the freeze drying process via acting as cryoprotectants. The freeze drying process involves two major steps: freezing followed by sublimation under reduced pressure, which in turn creates stresses on the dried formulation resulting in particle aggregation and breakdown. Having the ability to adsorb on the nanoparticle surfaces, cyclodextrins can restrict the motion of the freeze-dried nanoparticles. The literature includes several examples that illustrate the use of cyclodextrins as cryoprotectants and/or stabilizing agents for the preparation of freeze-dried nanoparticles as poly(D,L-lactide-co-glycolide) nanospheres [47], liposomes [48], PEGylated liposomes [49] and chitosan nanoparticles [50]. 

In the present study, terconazole-loaded silica/chitosan nanoparticles are prepared by a simple cross-linking between tetraethyl ortho silicate as a source of silica and chitosan HCl as a source of nitrogen. The applied fabrication method was performed at room temperature without commonly used aggressive conditions of temperature, acids and surfactants, which results in the production of regular-shaped nanoparticles. The freeze drying of the prepared nanoparticle dispersions were carried out using cyclodextrins as carriers. The prepared formulations were characterized for their particle size, zeta potential, percentage yield, drug loading percentage and in vitro drug release. Optimization was pursued using a full factorial experimental design. The optimized formulation was further investigated through Fourier transform infrared spectroscopy studies, differential scanning calorimetry and X-ray diffraction. Additionally, the morphological examination using scanning and transmission electron microscopes was performed. Finally, the biological performance of the optimized formulations was assessed in Albino rabbits.

The literature sheds light on several studies in which chitosan/TEOS were combined for several purposes, such as developing microparticles [51], hydrogels [52] and matrices [53]. The current study uses this combination for the preparation of nanoparticles without the need for any surfactants. Furthermore, the solidification of the silica/chitosan nanoparticle dispersion by freeze drying using cyclodextrins as cryoprotectants is addressed in the present study for the first time. 

## 2. Materials and Methods 

### 2.1. Materials

Terconazole (TZ) was gifted by Minapharm Pharmaceuticals (Cairo, Egypt). Tetraethyl ortho silicate (TEOS; 98%) and dialysis tubing cellulose membranes (molecular weight cut-off 12,000–14,000 g/mole) were acquired from Sigma-Aldrich (Saint louis, MO, USA). Methyl-β-cyclodextrin (MβCD) and hydroxylpropyl-β-cyclodextrin (HPβCD) were donated by Roquette (Beinheim, France). Chitosan hydrochloride was kindly brought from Zhejiang Chemicals Import and Export Corporation (Hangzhou, China). Sodium chloride, sodium dihydrogen phosphate monohydrate, disodium hydrogen phosphate heptahydrate and isopropyl alcohol were procured from El-Nasr Pharmaceutical Chemicals (Cairo, Egypt). Mucin from porcine stomach type II with bound sialic acids 1% was purchased from Sigma-Aldrich, Chemie GmbH (Tokyo, Japan). The rest of the chemicals and solvents were of analytical grade and were utilized as received. The water used was deionized, bi-distilled water. 

### 2.2. Methods

#### 2.2.1. Preparation of Terconazole-Loaded Silica/Chitosan Nanoparticles (SCNs)

Terconazole-loaded SCNs were prepared by the freeze-drying technique. An alcoholic drug solution was prepared by dissolving an accurate amount of terconazole in isopropyl alcohol under sonication (water bath sonicator; Elma S30H, Singen, Germany) for 5 min until a clear solution was obtained. In parallel, a clear aqueous solution was formed by dissolving accurately weighed amounts of chitosan HCl in water. Afterwards, the aqueous solution was carefully added, portion-wise, onto the alcoholic solution containing the drug under magnetic stirring (Stuart, SB162, Staffordshire, UK) at 400 rpm for 5 min. Subsequently, a specific amount of TEOS (5, 10 or 15% *w*/*v*) was added to the hydroalcoholic solution that was constantly stirred (400 rpm) at ambient temperature for 24 h. The concentrations of terconazole and chitosan HCl were 0.5 mg/mL and 0.1% *w*/*v*, respectively, in the final hydroalcoholic mixture.

For the preparation of the freeze-dried formulations, two types of cyclodextrins, hydroxypropyl-β-cyclodextrin (HPβCD) and methyl-β-cyclodextrin (MβCD), were used as cryoprotectants. The cryoprotectants were dissolved directly into the formulation, followed by freezing for 24 h at −80 °C in an ultra-low temperature deep freezer (Thermo Fisher Scientific ExF24086V, Waltham, MA, USA), prior to the freeze-drying process (Christ freeze dryer, ALPHA 2–4 LD plus, Osterode, Germany), which lasted for 48 h.

#### 2.2.2. Statistical Analysis of the Experimental Design, Evaluation and Optimization of the Prepared SCNs

The SCNs were prepared and optimized using a 2^3^.3^1^ full factorial experimental design to explore the influential effect of formulation variables on the produced nanoparticles using Design-Expert^®^ 7 software (Stat-Ease, Minneapolis, MN, USA). Four variables were chosen as the independent variables, three factors at two levels and one factor at three levels. The independent variables were: (A) the percentage of the aqueous phase, (B) the percentage of TEOS, (C) the percentage of cryoprotectant and (D) the cryoprotectant type, while the particle size (PS), polydispersity index (PDI), percentage yield, and drug loading (DL), percentage drug released after 2 h (Q_2_) and 6 h (Q_6_) were taken as the dependent variables. Optimum formulation was selected based on the maximizing percentage yield, DL and Q_6_ as well as minimizing PS and Q_2_ values. The composition of the freeze-dried SCNs is compiled in Table 1.

##### Determination of Particle Size (PS), Polydispersity Index (PDI) and Zeta Potential (ZP)

An accurate amount of the freeze-dried SCNs were dispersed in deionized distilled water (1:10 *w*/*v*) and their PS, particle size distribution (polydispersity index; PDI), and ZP values were measured using ZetaSizer Nano ZS (Malvern Instruments, Worcestershire, UK).

##### Determination of the Percentage Yield 

SCN powder collected from the freeze dryer was weighed and the % yield was determined by applying the following equation:

(1) Yield (%)=(Recovered SCNs weight/Total initial solids weight)×100

##### Drug Loading (DL)

The DL for the prepared SCNs was determined and compared. The definite weights from each formulation were dissolved in isopropyl alcohol to extract the loaded drug and determine its concentration (drug content) spectrophotometrically (UV Spectrophotometer, model UV-1601 PC; Shimadzu, Kyoto, Japan) at the predetermined λ_max_ 245.2 nm. The DL was calculated as follows:

(2) $$\mathrm{DL}\;\left(\%\right)=\frac{\mathrm{Drug}\;\mathrm{content}}{\;\mathrm{Weight}\;\mathrm{of}\;\mathrm{nanoparticles}}\times 100$$

##### In Vitro Drug Release Studies

The release pattern of the prepared SCNs was examined using the dialysis bag method [54]. Briefly, an accurately weighed amount from the freeze-dried particles, equivalent to 1 mg drug, were suspended in 1 mL of isotonic phosphate buffer, pH 7.4, and instilled into the dialysis bag (soaked in distilled water for 12 h in advance) representing the donor compartment, and immersed in 20 mL of isotonic phosphate buffer, pH 7.4, representing the receptor compartment. All the systems were kept at 32 ± 0.5 °C in an incubation shaker (IKA KS 4000, Staufen, Germany) stirred at 160 rpm. At pre-certain time intervals, 3 mL aliquots of the release medium were withdrawn and substituted by new medium. The withdrawn samples were analyzed for terconazole content spectrophotometrically at 245.2 nm. The release of drug suspension was carried out following the same procedures for comparison purposes.

The percentage mean cumulative drug release was illustrated against time, and the % drug released after 2 and 6 h, (Q_2_ and Q_6_, respectively) was calculated for the prepared formulations.

### 2.3. Preparation of the Modified Terconazole-Loaded Silica/Chitosan Nanoparticles (SCNs)

Modified terconazole SCNs were fabricated via the freeze-drying technique following the same procedures previously mentioned under Section 2.2.1, but with minor modifications, where the nominal drug concentration was raised from 0.5 to 1 mg/mL in the final hydroalcoholic mixture used to prepare the optimized formulation SCM8. The modified formulation was coded as SCM8-1.

The effect of increasing the nominal drug concentration on formulation SCM8-1 properties (PS, PDI, yield, ZP, DL and in vitro release behavior) was assessed by applying the same procedures as previously mentioned.

#### 2.3.1. Characterization of the Modified Terconazole-Loaded Silica/Chitosan Nanoparticles (SCNs)

##### Fourier Transform Infrared Spectroscopy (FTIR)

The FTIR spectra were recorded for the modified non-medicated SCM8-1, its non-medicated physical mixture as well as TEOS, chitosan HCl and MβCD using FTIR spectrophotometer Model 22, Bruker, Billerica, MA, U.K., operated by the KBr disk technique. The FTIR measurements were performed in the scanning range of 4000–400 cm^−1^ at ambient temperature.

##### Differential Scanning Calorimetry (DSC)

The thermal performance of the modified formulation was conducted using DSC analysis (DSC-50, Shimadzu, Japan). Thermograms of the modified formulation and the individual components were investigated.

Certain weights from each sample (5 mg) were heated in aluminum pans individually at a heating rate of 10 °C/min, over a temperature range of 30 to 400 °C.

##### X-ray Diffraction Studies (XRD)

X-ray diffraction patterns of the modified formulation along with its individual ingredients were recorded. The samples were irradiated with Ni filtered Cu Kα radiation, with an operating voltage of 45 kV and a current of 40 mA using Diano X-ray diffractometer apparatus (Woburn, MA, USA). The employed scanning rate was 2°/min over a diffraction angle (2θ) range of 3–70°.

##### Scanning Electron Microscopy (SEM)

The morphology and surface properties of the modified formulation were evaluated using SEM (Quanta FEG250, Hillsboro, OR, USA). The SEM was operated at 20 kV. The samples were first prepared for scanning by sputtering gold using a Poloron DC “sputtering unit” operated at 1.4 kV and 18–20 mA.

##### Transmission Electron Microscopy (TEM)

The modified formulation was diluted with deionized bi-distilled water. Subsequently, the sample was stained with 1% of phosphotungstic acid solution and then dried at ambient temperature to be scanned at an accelerating voltage of 80 kV via TEM (JEM-HR-2100, Jeol, Tokyo, Japan). 

##### Mucoadhesion Study

To study the mucoadhesive properties of the modified formulation, a mucoadhesion study was performed. In brief, a mucin aqueous solution (0.4 mg/mL) was prepared by dissolving accurately weighed amounts of mucin in distilled deionized water under the effect of a vortex. Then, the selected formulation suspended in deionized water was mixed with an equivalent volume of the prepared mucin solution (1:1 *v*/*v*) for 1 min by vortexing. The resulting mixtures were assessed for their ZP and PS values using ZetaSizer Nano ZS (Malvern Instruments, Worcestershire, UK) [55,56].

#### 2.3.2. In Vivo Histopathological Studies

Histopathological studies were conducted to assess the safety of the prepared SCNs following long-term ocular therapy. Three male Albino rabbits (2.5–3 kg) were given three daily doses of the modified formulation in the right eye every day for a one week, where the left eye served as a control (untreated). On the seventh day, the rabbits were decapitated under mild anesthesia. Following this, the eyeballs were separated, cleaned with a saline solution and directly fixed with a 10% formal saline solution for 24 h. After fixation, dehydration was performed by employing repeated dilutions of ethyl alcohol. The dehydrated samples were cleaned in xylene and embedded in paraffin at 56 °C in a hot air oven for 24 h. Paraffin blocks were cross-sectioned at a thickness of 4 microns by a sledge microtome. The acquired cross sections were mounted on glass slides, de-paraffinized and stained by hematoxylin and eosin stain to be routinely observed under a light electric microscope [57].

#### 2.3.3. In Vivo Evaluation of the Selected Terconazole-Loaded Silica/Chitosan Nanoparticles (SCNs)

##### Study Design

The tested formulations were sterilized at the irradiation facility at the National Centre for Radiation Research and Technology (NCRRT) in the Egyptian Atomic Energy Authority (Cairo, Egypt) within a cobalt-60 gamma chamber 4000-A, using gamma radiation at a dose of 10 kGy prior to investigation. The modified formulation was investigated for its biological performance compared to the drug suspension. Briefly, the selected formulation as well as the crude drug were suspended in an isotonic phosphate buffer, pH 7.4, to prepare samples equivalent to 1 mg/mL of terconazole. The study design was approved by the Research Ethics Committee, the Faculty of Pharmacy, Cairo University, Cairo, Egypt (03/2020 PI 2652). Sixteen healthy male Albino rabbits (weighing 2.5–3 kg) were selected for the study and were randomly divided equally into two groups. Each group was divided into two subgroups (*n* = 4). A non-blind, parallel design was applied to the investigated samples, where each group received a single ocular dose of the tested formulation or the drug suspension. A volume of 50 μL of the investigated samples was instilled into the conjunctival sac of the eye. 

##### Tear Film Sampling

At each determined time point, tear samples were collected from four rabbits (*n* = 4) and then the experiment was repeated on new rabbits to collect the tear samples at another time point. To collect the tear samples, three sterile filter paper discs (6 mm in diameter) were placed under the lower eyelid of the rabbit’s eye for only 30 s at the following time points: 0.5, 1, 2, 4, 6, 9 and 24 h. During the sampling process, care was taken to prevent irritating the eyelid margin. The discs were collected and soaked in 100 µL of ethanol before being stored in Eppendorf tubes at −20 °C until they were analyzed.

##### Chromatographic Conditions

The concentration of terconazole was analyzed in the collected rabbits’ tear films by applying a validated LC-MS/MS technique (Shimadzu, Kyoto, Japan) equipped with a triple quadrupole mass spectrometer (API 4000, AB Sciex Instruments, Framingham, MA, USA). Dapoxetine was utilized as an internal standard. The mobile phase consisted of 80% acetonitrile, 20% H_2_O and 0.1% formic acid, and its pH value was adjusted at 8.5. 

A sample volume of 15 μL was injected into the column and the elution was adjusted at a flow rate of 1.5 mL/min. The separation was conducted on a C_18_ reversed phase analytical column (250 mm × 4.6 I.D. mm, particle size 5 μm, Berlin, Germany) and the drug was detected at 220 nm. 

The pharmacokinetic parameters of terconazole in the tear film were calculated using WinNonlin^®^ software (version 3, Scientific Consulting Inc., Cary, NC, USA). The values for the maximum tear concentration and its time (C_max_ and t_max_, respectively), as well as the area under the tear concentration–time curve up to 24 h (AUC_0–24_), were determined for the tested formulations. One-way analysis of variance (ANOVA) was conducted to statistically analyze the pharmacokinetic data.

## 3. Results and Discussion 

### 3.1. Preparation of the Terconazole-Loaded Silica/Chitosan Nanoparticles (SCNs)

Nanoparticles are promising ocular dosage forms that have the capability of enhancing the delivery and efficacy of poorly water-soluble drugs, such as terconazole [58,59]. Silica nanoparticles represent a versatile and efficient nano-platform. In this study, silica nanoparticles were synthesized via the crosslinking between TEOS and chitosan HCl, representing the silica and nitrogen sources, respectively. Isopropyl alcohol was used as a solvent for terconazole as it is miscible with both TEOS and the aqueous solution. This method depends on the hydrolysis of TEOS in the presence of water and alcohol, and then the condensation reaction between the hydrolyzed TEOS and chitosan HCl to form the nanoparticles. The formation of nanoparticles was detected by the formation of a slightly turbid translucent solution by time. 

Different drug-loaded SCNs were prepared by varying their composition. The concentration of both the drug and chitosan HCl was kept constant throughout the study (0.5 mg/mL and 0.1 %*w*/*v*, respectively); however, the percentage of the aqueous phase relative to the total volume of the preparation mixture (hydroalcoholic solution) was varied. 

It was observed that the freeze-dried SCNs prepared without the use of cryoprotectants formed aggregates that were difficult to re-disperse. This might be attributed to the desiccation stresses generated during the freeze-drying process. Cyclodextrins were used as cryoprotectants for their ability to act as solubilizing and stabilizing agents [60,61]. Two types of cyclodextrins (MβCD and HPβCD) were used as cryoprotectants for the nanoparticles during the freeze-drying process. 

All the formulations were successfully prepared at room temperature, introducing no austere conditions compared to those commonly used in silica nanoparticle preparation in the literature. 

### 3.2. Statistical Analysis of the Experimental Design, Evaluation and Optimization of the Prepared SCNs

To optimize the prepared SCNs, a 2^3^.3^1^ full factorial design was applied. The (A) % of aqueous solution, (B) % TEOS, (C) % cryoprotectant and (D) cryoprotectant type were chosen as the formulation variables, while the PS, PDI, % yield, %DL, Q_2_ and Q_6_ were used as the responses. The responses of the prepared formulations are shown in Table 1. 

Both the independent variables and responses were related using a polynomial equation with statistical analysis through Design-Expert^®^ 7 software.

#### 3.2.1. Effect of the Formulation Factors on the Particle Size (PS) and Polydispersity Index (PDI)

The PS values of the investigated SCN formulations ranged from 316.33 ± 28.02 to 594.00 ± 28.28 nm for SCM5 and SCH7, respectively, while the PDI values were between 0.224 ± 0.017 and 0.767 ± 0.097 for SCH6 and SCM7, respectively, as shown in Table 1. The results of the ANOVA test show that two independent factors, (A) the percentage of the aqueous phase and (B) the percentage of TEOS, had a significant (*p* = 0.0001 and *p* = 0.0002, respectively) effect on the nanoparticle PS values and also on the PDI values (*p* = 0.0001 for both factors).

**Figure 1:**
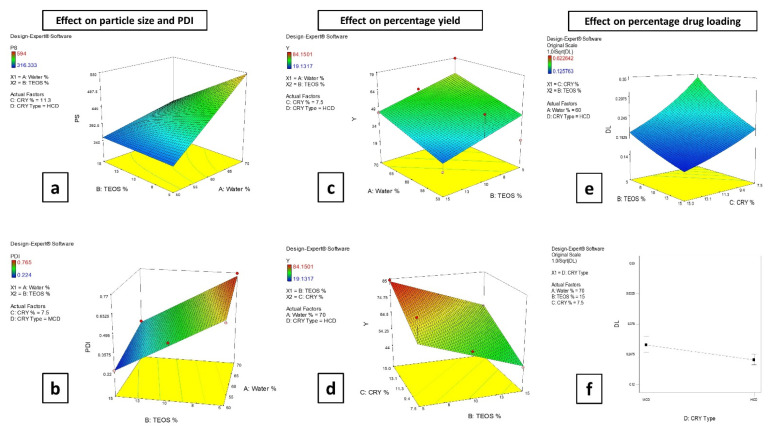
Response surface plots showing the effect of the aqueous phase percentage (Water %) and TEOS percentage (TEOS %) on particle size; PS (**a**), on polydispersity index; PDI (**b**) and on percentage yield; Y (**c**). The effect of TEOS percentage (TEOS %) and cryoprotectant percentage (CRY%) on percentage yield; Y (**d**) and on percentage drug loading; DL (**e**). The effect of cryoprotectant type (CRY Type) on percentage drug loading; DL (**f**).

According to the ANOVA results, increasing the percentage of the aqueous phase from 50 to 70% in the preparation results in a significant increase in the PS values of the prepared SCNs (Figure 1a). This might be attributed to the increase in the immiscibility gap of the TEOS–hydroalcoholic mixture and thus less hydrolysis of TEOS [62], leading to the formation of larger nanoparticles (nanoparticle growth). As presented in Figure 1b, a significant increase in the PDI values of the SCNs was obtained with an increasing water concentration.

On the other hand, increasing TEOS percentage from 5 up to 15% in the preparation resulted in a significant decrease in PS (Figure 1a). This decrease in nanoparticle PS values might be due to the availability of a larger amount of TEOS molecules, which hydrolyzed to form more nuclei that interacted faster with chitosan HCl (condensation) to form nanoparticles with a smaller PS. Qi et al. found that by increasing the TEOS amount, the hydrolysis rate predominated the condensation rate during the reaction, leading to the formation of particles with smaller PS values [63]. Furthermore, the PDI values of the silica nanoparticles were reduced upon increasing the TEOS concentration (Figure 1b).

#### 3.2.2. Effect of the Formulation Factors on the Percentage Yield

The yield values of the investigated SCN formulations ranged from 19.36 ± 0.32 to 84.42 ± 0.37%, as shown in Table 1. The results of the ANOVA test show that three independent factors, (A) the percentage of aqueous phase, (B) the percentage of TEOS and (C) the cryoprotectant percentage, had a significant effect (*p* = 0.0007, *p* = 0.0029 and *p* = 0.0008, respectively) on the nanoparticle yield values. As illustrated in Figure 1c, increasing the percentage of the aqueous phase from 50 to 70% *v/v*, resulted in a significant increase in the nanoparticle yield values. Furthermore, increasing the TEOS percentage from 5 to 10 or to 15% in the preparation, results in a decrease in the nanoparticle yield values, as presented in Figure 1c. 

From the previous collected data for the particle size and yield values, we can determine that the higher the obtained size for the nanoparticles, the higher the yield for these nanoparticles. This can be explained by the low weight “fluffiness” for smaller particles, compared to larger ones that may cause their loss during preparation and handling.

Finally, it can be observed that increasing the cryoprotectant percentage (regardless of its type) from 7.5 to 15% in the preparation, results in an increase in the nanoparticle yield values, as shown in Figure 1d. This might be due to increasing the total solid content in the final freeze-dried preparation.

#### 3.2.3. Effect of the Formulation Factors on the Drug Loading (DL)

The DL values of the investigated SCN formulations range from 0.13 ± 0.00 to 0.63 ± 0.01% (Table 1). The ANOVA test results revealed that three of the independent factors, (B) the percentage of TEOS, (C) the percentage of cryoprotectant and (D) the cryoprotectant type, had a significant (*p =* 0.0003, *p* < 0.0001 and *p* = 0.0204, respectively) effect on the DL values. 

The results confirm that increasing the TEOS level from 5 to 15% in the preparation results in a significant decrease in the nanoparticle DL values, as shown in Figure 1e. This decrease in DL values might be attributed to the enhanced crosslinking between TEOS molecules and chitosan molecules during the formation of nanoparticles, as previously mentioned in Section 3.2.2.

It is evident that increasing the cryoprotectant percentage, irrespective of its type, from 7.5 to 15% resulted in a significant decrease in the nanoparticle DL values, as presented in Figure 1e. This might be attributed to the increased yield values of the preparation [64,65].

In addition, substituting MβCD with HPβCD in the formulation significantly decreases the nanoparticle DL values at all the studied factors levels (Figure 1f). Both MβCD and HPβCD are derivatives for the parent βCD prepared by its substitution with methyl and hydroxyl propyl groups, respectively. Hydroxyl propyl groups are bulky groups of high steric impediment, and hence might hinder the attachment of the lipophilic part of terconazole to the hydrophobic cavity of HPβCD [66].

#### 3.2.4. Effect of the Formulation Factors on Q_2_ and Q_6_

Approximately 70% of the drug solution was released from the cellulose membrane after 3 h, while only 9% of the drug was released in case of drug suspension in the same period. As presented in Table 1, it can be observed that Q_2_ values range from 8.50 ± 0.08 to 27.10 ± 0.30%, where the highest value can be observed for formulation SCM6, whereas formulation SCH2 shows the lowest Q_2_ value. On the other hand, formula SCM1 shows the highest Q_6_ value (52.51 ± 0.42%), while formula SCH12 exhibits the lowest Q_6_ value of 14.75 ± 0.40%. The results indicate that (B) the TEOS percentage has a significant (*p* = 0.0160) effect on the Q_2_ values. Additionally, (C) the percentage of cryoprotectant and (D) cryoprotectant type show significant (*p* = 0.0030 and *p* = 0.0029, respectively) effects on the Q_2_ values, in addition to their significant (*p* = 0.0014 and 0.0001, respectively) effect on the Q_6_ values. 

**Figure 2:**
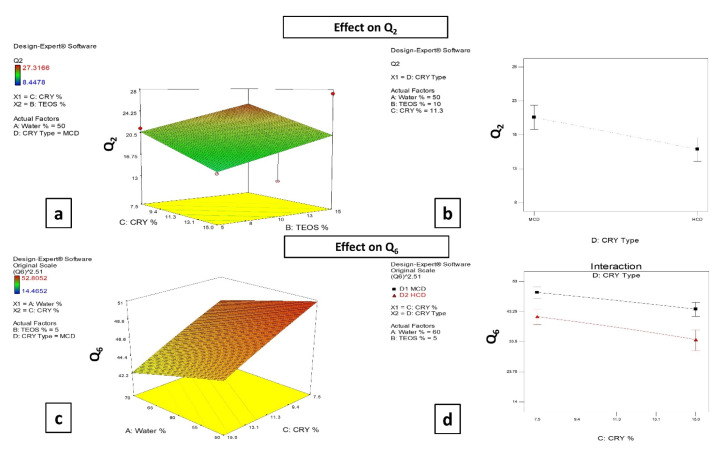
Response surface plots showing the effect of: TEOS percentage (TEOS %) and cryoprotectant percentage (CRY %) on Q_2_ (**a**) and effect of cryoprotectant type (CRY Type) on Q_2_ (**b**). The effect of aqueous phase percentage (Water %) and cryoprotectant percentage (CRY %) on Q_6_ (**c**) and effect of cryoprotectant percentage (CRY %) and cryoprotectant type (CRY Type) on Q_6_ (**d**).

It was observed that increasing TEOS percentage from 5% to 15% resulted in a significant increase in Q_2_ values, which might be ascribed to the smaller PS obtained with increasing TEOS concentration, as previously mentioned. The surface area-to-volume ratio increases as the PS becomes smaller. As a result, compared to a larger particle, more drug molecules would be allocated closer to the particle surface. Being at or near the surface results in a quicker drug release [67,68], as shown in Figure 2a. 

Increasing the cryoprotectant concentration from 7.5% to 15% causes a significance decrease in Q_2_ (Figure 2a) and Q_6_ (Figure 2c) values. Changing the cryoprotectant type from MβCD to HPβCD causes a significant decrease in both Q_2_ (Figure 2b) and Q_6_ (Figure 2d) values. Referring to the nanoparticle DL value results and by relating the associated decrease in DL with the increasing the cryoprotectant concentration, it could be deduced that the driving force for the drug to be released would decrease, resulting in a more sustained release pattern for the drug, which is in accordance to Fick’s first law [69,70]. Similar explanations were reported when changing the cryoprotectant type.

The proposed scenario for the obtained sustained drug release might be endorsed by many factors. The incorporation of the drug inside the formed nanoparticles might explain the retarded drug release from the nanoparticles. This might be ascribed to the formation of channels containing a saturated solution of the drug after coming into contact with the release medium, hence resulting in slower drug diffusion. Additionally, the hydrophobic nature of the drug as well as its suitable size enabled it to replace the water molecules inside the cyclodextrin cavity, making the release of the drug from the cavity a sustained process [71]. Another assumption might be due to the possible combination between the cyclodextrins and surface of the nanoparticles via hydrogen bonding, which might retard drug (either free and/or that released from the nanoparticles) release through the formation of a denser matrix. This combination between cyclodextrin molecules and nanoparticles might account for the successful role of the added cyclodextrins as cryoprotectants. 

All the prepared formulations possessed positive ZP values ranging from 12.65 ± 0.21 to 39.00 ± 2.59 mV. Moreover, the calculated drug content values were between 68.67 ± 0.46 and 108.17 ± 0.04%. 

**Figure 3:**
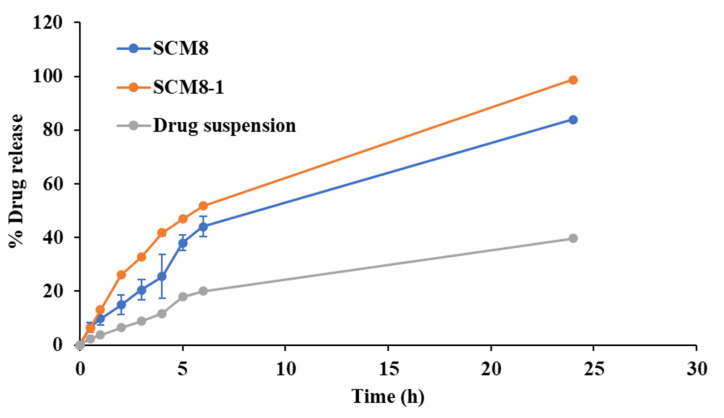
Release profiles of terconazole from SCM8 and SCM8–1 formulations, compared to the drug suspension.

Based on the obtained results and applying the desirability function, the formulation SCM8, having the maximum DL, Q_6_ and minimum PS and Q_2_ values, was selected for further modification to increase its drug loading percentage. A different drug loading value (1 mg/mL) was attempted in the modified formulation (SCM8-1). The results reveal a non-significant change (*p* > 0.05) in the % yield of SCM8-1 compared to the unmodified formulation SCM8 (82.34 ± 0.23 vs. 79.61 ± 2.29%). However, a significant increase in PS and DL values *(p* < 0.05) were observed, indicating the efficient entrapment of the drug inside the prepared particles. Additionally, a significant rise in Q_2_ and Q_6_ values (*p* < 0.05) can be observed in their release profiles (Figure 3), which denotes that the distribution of the loaded drug within the nanoparticles influences the release profile of the drug, with enhanced Q_2_ values suggesting the adsorption of a larger portion of the loaded drug on the surface of the nanoparticles, followed by the slow release phase representing the entrapped part within the nanoparticles [72]. The recorded values of PS were 538.60 ± 33.15 vs. 718.55 ± 9.83 nm, while the DL values were calculated to be 0.30 ± 0.01 vs. 0.61 ± 0.02 %, the Q_2_ values were increased from 14.44 ± 0.64 to 25.90 ± 0.55% and the Q_6_ value increased from 44.60 ± 0.69 to 51.44 ± 3.33% for SCM8 and SCM8-1, respectively. 

The obtained PDI was 0.60 ± 0.01 and ZP was 38.75 ± 0.35 mV for SCM8-1. The low PDI values indicate the narrow particle size distribution and formation of more stable nanoparticles [73]. Moreover, high ZP values were favored due to electric repulsion and, thus, less particles aggregation [74]. 

### 3.3. Characterization of the Modified Terconazole-Loaded Silica/Chitosan Nanoparticles (SCNs)

#### 3.3.1. Fourier Transform Infrared Spectroscopy (FTIR)

**Figure 4:**
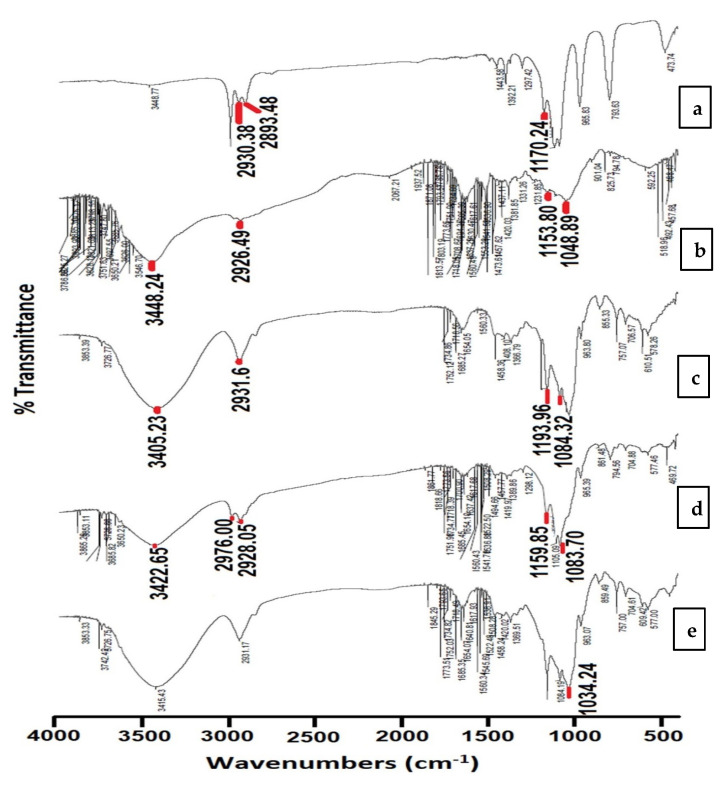
FTIR spectra for (**a**) TEOS, (**b**) chitosan HCl, (**c**) MβCD, (**d**) non-medicated physical mixture of SCM8–1 as well as (**e**) non-medicated SCM8–1.

Infrared spectroscopy is performed as a standard method for the structural characterization of the chemical entities and interactions between the used components in the formulation, as shown in Figure 4. The FTIR spectrum for TEOS showed absorption bands at 2930.38 and 2893.48 cm^−1^, which was due to the C–H aliphatic stretching, while the band showing 1170.24 cm^−1^ was due to the stretching vibrations of the C–O group. The chitosan HCl spectrum revealed different bands: one broad band at 3448.24 cm^−1^ due to the stretching of both NH_2_ and OH groups, and also stretching vibrations found at 1153.80 and 1048.89 cm^−1^ caused by the C–O group. The bending vibration located at 2926.49 cm^−1^ was caused by the aliphatic C–H group. 

The spectrum of MβCD showed a wide, broad band at 3405.23 cm^−1^ of the OH group and the stretching vibrations of the aliphatic C–H group at 2931.66 cm^−1^, while the characteristic bands of MβCD are those located at 1193.96 and 1084.32 cm^−1^. 

The FTIR spectrum of the non-medicated formula SCM8-1 showed a characteristic peak at 1034.24 cm^−1^ which is related to the chemical bonding between silica and nitrogen (Si–N single bonding). The absence of this peak in the FTIR spectrum of the non-medicated physical mixture of SCM8-1 confirmed that our method of preparation allowed for the interaction between TEOS and chitosan HCl, and the formation of this new Si–N bond [75]. This bond confirmed the condensation reaction between the Si groups of TEOS (obtained from hydrolysis of Si–O–CH_2_–CH_3_ groups) and C–NH_2_ group of chitosan HCl to form silica/chitosan nanoparticles.

#### 3.3.2. Differential Scanning Calorimetry Study

**Figure 5:**
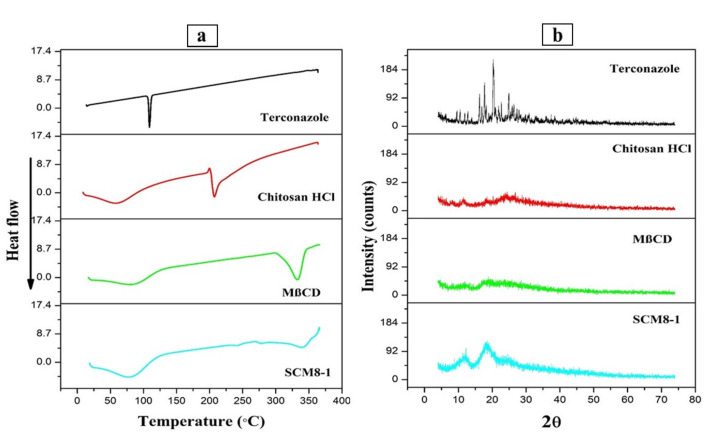
(**a**) DSC curves and (**b**) X–ray diffractions for terconazole, chitosan HCl, MβCD and the selected formulation SCM8–1.

The DSC thermograms of formulation SCM8-1 and its components are displayed in Figure 5a. The DSC thermogram of pure terconazole shows a single sharp endothermic peak at 126.21 °C due to its melting, and represents its crystalline nature. A chitosan HCl thermogram revealed two endothermic peaks: a broad one at 92.93 °C corresponding to water loss, and an exothermic peak at 218.11 °C indicating its degradation. Pure MβCD displayed a broad endothermic peak at 84.09 °C due to moisture loss and another one at 332.80 °C owing to decomposition. 

The thermogram of the formulation revealed that the distinctive peaks for its components had completely disappeared, which may be attributed to the component’s molecular dispersion in the matrix system of the produced nanoparticles.

#### 3.3.3. X-ray Diffraction Study (XRD)

The X-ray diffraction study was performed to characterize the physical state of the drug, either crystalline or amorphous, before and after formulation. X-ray diffractograms of formulation SCM8-1 as well as its physical mixture and individual components are displayed in Figure 5b. The diffraction spectrum of the pure drug represented several distinctive sharp peaks at 16.28°, 17.72°, 20.54°, 20.25° and 24.89° (2θ), indicating its crystalline nature. The diffraction patterns of chitosan HCl and MβCD showed typical halo patterns, which are characteristic for the amorphous state. The powder XRD of SCM8-1 showed wide diffractograms due to the amorphous nature of the formed nanoparticles that also confirms the results obtained by the DSC study.

#### 3.3.4. Scanning Electron Microscopy (SEM)

**Figure 6:**
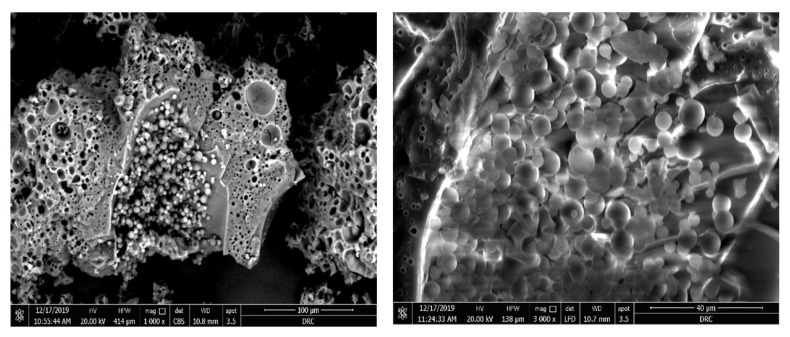
SEM Images for the SCM8–1 formulation showing the regular-shaped particles.

The SEM image (Figure 6) for the modified formulation (SCM8-1) shows spherical particles with a smooth surface embedded in the cryoprotectant matrix, indicating the successful action of the added cryoprotectant in preserving the nanoparticles during exposed stresses in the freeze drying process. There were no drug crystals detected on the nanoparticles’ surfaces, indicating that the drug was amorphized within the nanoparticle’s matrix.

#### 3.3.5. Transmission Electron Microscopy (TEM)

**Figure 7:**
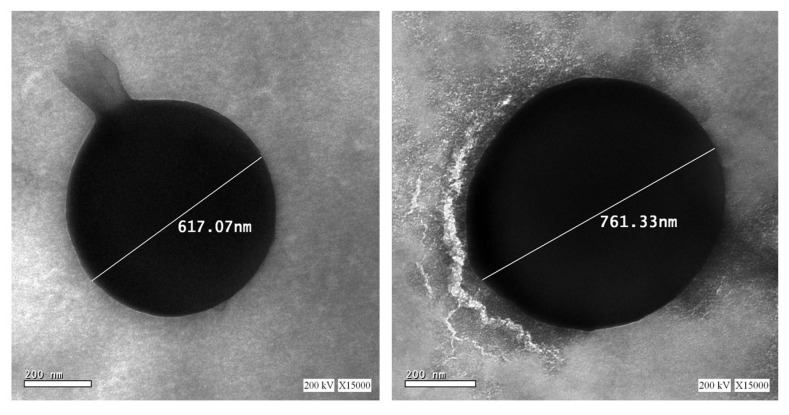
TEM images SCM8–1 formulation showing the spherical-shaped particles with their particle size values (scale bar: 200 nm).

The TEM images for the modified formulation (SCM8-1) present well-dispersed spherical particles with a PS range of 610–770 nm, as shown in Figure 7. The PS range obtained from TEM was in good agreement with that obtained from ZetaSizer. 

#### 3.3.6. Mucoadhesion Study

Mucins are hydrophilic components essential for protecting the ocular epithelia. Mucins contribute to the efficient lubrication and wettability of the ocular surface [76]. Hence, targeting the mucins (negatively charged due to the presence of sialic groups) through ionic interaction with positively charged groups [77] is highly recommended to extend the residence time of the formulation, and in turn improve drug efficacy and bioavailability.

**Figure 8:**
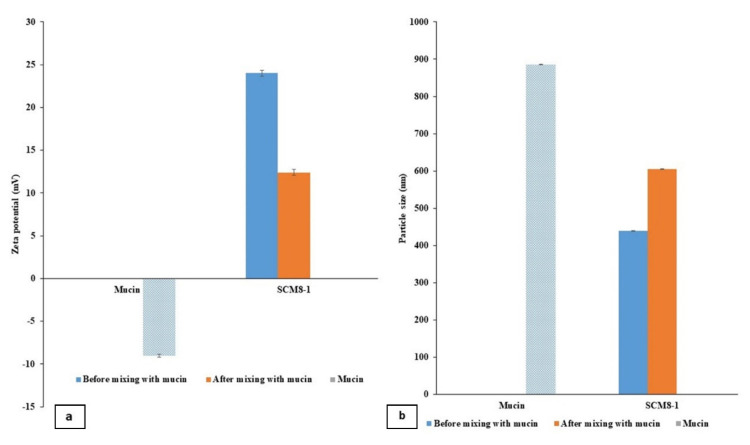
(**a**) Zeta potential and (**b**) particle size values of the selected formulation SCM8–1, before and after mixing with mucin solution.

This ionic interaction is clarified in Figure 8a, showing the effect of adding a mucin solution to the ZP value of the examined formulation SCM8-1. A significant decrease (*p* < 0.05) in the ZP value after adding the mucin solution from 26.7 ± 0.05 to 12.4 ± 0.03 mV was observed. A further confirmation for this ionic interaction was noticed with the significantly increased PS (*p* < 0.05) of SCM-8 after mixing with the mucin solution, compared to the unmixed formulation. The obtained PS values after mixing with the mucin solution, compared to the values before mixing, were found to be 655.8 ± 0.34 vs. 493.7 ± 0.56 nm, as shown in Figure 8b. This observed decrease in the ZP value, along with the increase in PS values, indicated the mucoadhesive properties of SCNs generated from the interaction of positively charged chitosan groups with negatively charged mucin. 

### 3.4. Ocular Irritation Study

**Figure 9:**
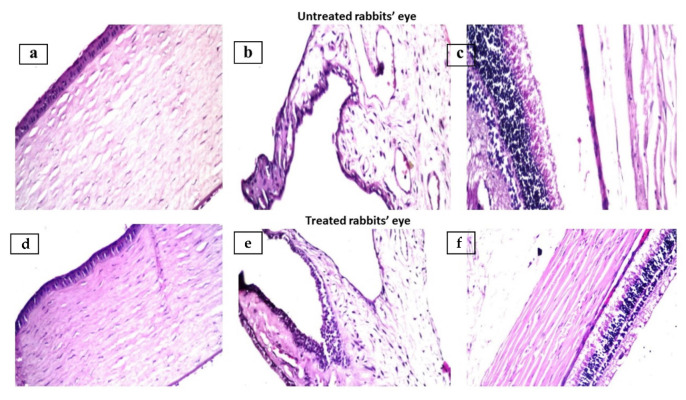
Photomicrographs showing histopathological sections of normal untreated and treated rabbits’ eyes (SCM8–1 formulation) stained with hematoxylin and eosin. The photomicrographs show histological structure of the (**a**,**d**) cornea, (**b**,**e**) iris, (**c**,**f**) retina, choroid and sclera (×40).

The safety and compatibility of ocular medicines are prerequisites for a successful ocular drug delivery. That is why the chosen formulation, SCM 8-1, was assessed for its histopathological behavior in male Albino rabbits. The stained sections of cornea, iris, retina, choroid and sclera of the treated and control eyes are presented in Figure 9. By comparing the two groups, no histopathological abnormalities could be detected in the examined sections, indicating the preservation of the eyeball structure, even after intensive application of the formulation. This evidence indicates the safety and biocompatibility of terconazole-loaded SCNs as an ocular delivery system. Several studies discussed the safety of silica particles [78,79], chitosan HCl [39,80,81] and cyclodextrins [82,83,84] for ocular delivery. Moreover, the results reveal that the used concentrations are suitable and safe.

### 3.5. In Vivo Evaluation of the Selected Terconazole-Loaded Silica/Chitosan Nanoparticles (SCNs)

**Figure 10:**
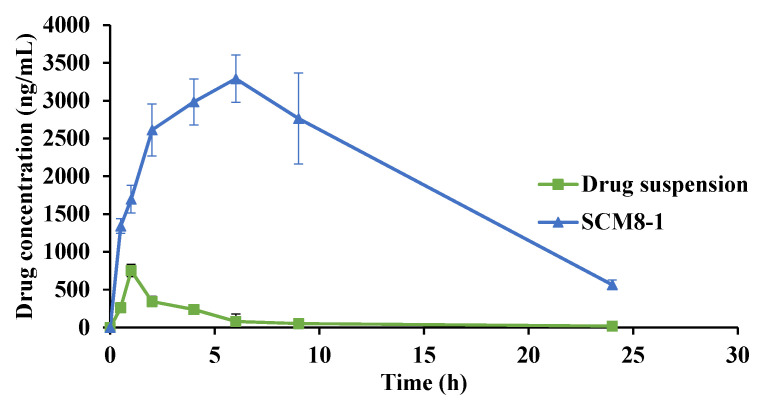
Mean terconazole tear concentrations following the ocular administration of SCM8-1 formulation compared to drug suspension.

The pharmacokinetic parameters of terconazole were calculated for the modified formulation, SCM8-1, and compared to the drug suspension after dosing in rabbits’ eyes at a concentration of 1 mg/mL. As presented in Table 2 and Figure 10, it is evident that both samples show completely different tear concentration/time curve profiles with the superiority of the examined formulation (*p* < 0.05) over the drug suspension regarding the C_max_, t_max_ and AUC_0–24_ values. The C_max_ values for formulation SCM8-1 and drug suspension were 3449.25 ± 148.57 and 752.19 ± 81.17 ng/mL, achieved after 7 and 1 h, respectively. Regarding the C_max_ and AUC_0–24_ values, it was found that a 4.6- and 19.7-fold increase, respectively, was obtained with formulation SCM8-1 compared to the drug suspension.

This achieved enhancement in the ocular bioavailability of terconazole from formulation SCM8-1, in addition to the prolonged residence time in the eye (a greater MRT value compared to drug suspension), might be ascribed to the mucoadhesive properties of the formulation, due to the cationic structure of chitosan, which was responsible for the prolonged corneal residence time as well as better permeability due to its ability to temporarily open the tight junctions [85,86,87]. Additionally, the formulation of the drug in the form of nanoparticles might improve drug penetration via overcoming the ocular barriers, resulting in the achievement of optimum concentrations of the loaded drug at the site of action [88,89]. Cyclodextrins have the ability to solubilize lipophilic drugs, such as terconazole, as well as augment drug permeability through ocular tissues, resulting in improved ocular bioavailability [56]. 

## 4. Conclusions

Silica/chitosan nanoparticles were fabricated through the crosslinking between TEOS and chitosan HCl under magnetic stirring at room temperature, followed by freeze drying utilizing cyclodextrins as cryoprotectants. The chemical interaction between both components was detected by FTIR spectra. The prepared nanoparticles succeeded to sustain the drug release compared to pure drug solution, and thus enhanced the compliance of the patients. The optimized formulation had minimum drug release at 2 h, as well as the best results for drug loading. However, a better enhancement in % drug loading was achieved by modifying the optimized formulation via increasing the nominal drug concentration. The modified formulation showed well-dispersed spherical particles with a particle size in the nano-range. Having chitosan HCl in the composition of the formed nanoparticles imparted a positive charge on the surface of the particles, which offered the nanoparticles excellent mucoadhesive properties. In vivo studies on male Albino rabbits emphasized the success of the constructed nanoparticles, in comparison to the drug suspension regarding enhanced C_max_, AUC_0-24_ values as well as more sustained action. The developed formulation introduced a successful ocular nanocarrier for hydrophobic drugs. Further optimization for the developed formulation as well as conducting stability studies should be carried out. 

## Figures and Tables

**Table 1:** Experimental runs and responses obtained for the factorial design of the freeze-dried terconazole-loaded silica/chitosan nanoparticles.

Code *	Independent Variables				Responses				
	Water (% *v*/*v*) in the Hydroalcoholic Solution	TEOS (%*w*/*v*)	Cryoprotectant Type; Percentage (%*w*/*v*)	Particle Size (PS; nm)	PDI	Yield (%)	Drug Loading (DL; %)	Q_2_ (%)	Q_6_ (%)
SCM1	50	5	MβCD; 7.5	399.50 ± 2.12	0.621 ± 0.012	30.44 ± 0.57	0.48 ± 0.01	21.92 ± 0.94	52.51 ± 0.42
SCM2	50	5	MβCD; 15	322.80 ± 37.68	0.634 ± 0.014	70.38 ± 0.36	0.24 ± 0.02	15.97 ± 0.40	43.62 ± 0.54
SCM3	50	10	MβCD; 7.5	329.50 ± 38.32	0.458 ± 0.009	57.36 ± 0.34	0.27 ± 0.03	19.66 ± 0.83	46.78 ± 0.70
SCM4	50	10	MβCD; 15	353.67 ± 41.67	0.465 ± 0.002	68.36 ± 0.23	0.21 ± 0.01	13.88 ± 0.35	43.69 ± 0.80
SCM5	50	15	MβCD; 7.5	316.33 ± 28.02	0.229 ± 0.009	23.48 ± 0.11	0.28 ± 0.03	23.32 ± 0.43	46.68 ± 0.89
SCM6	50	15	MβCD; 15	376.50 ± 7.78	0.238 ± 0.012	41.71 ± 0.21	0.19 ± 0.01	27.10 ± 0.30	51.63 ± 0.74
SCM7	70	5	MβCD; 7.5	560.14 ± 38.13	0.767 ± 0.097	62.55 ± 0.55	0.63 ± 0.01	21.34 ± 0.30	50.77 ± 0.71
SCM8	70	5	MβCD; 15	538.60 ± 33.15	0.747 ± 0.006	82.34 ± 0.23	0.30 ± 0.01	14.44 ± 0.64	44.60 ± 0.69
SCM9	70	10	MβCD; 7.5	451.33 ± 57.28	0.518 ± 0.008	58.38 ± 0.37	0.34 ± 0.02	22.42 ± 0.65	48.68 ± 0.50
SCM10	70	10	MβCD; 15	455.67 ± 60.87	0.551 ± 0.025	70.35 ± 0.32	0.20 ± 0.01	13.97 ± 0.33	38.31 ± 0.35
SCM11	70	15	MβCD; 7.5	363.00 ± 44.44	0.341 ± 0.007	42.44 ± 0.39	0.23 ± 0.01	24.99 ± 0.94	45.32 ± 0.09
SCM12	70	15	MβCD; 15	344.50 ± 41.33	0.319 ± 0.006	61.47 ± 0.19	0.18 ± 0.01	15.17 ± 0.25	40.14 ± 0.23
SCH1	50	5	HPβCD; 7.5	383.50 ± 21.92	0.633 ± 0.014	23.43 ± 0.40	0.36 ± 0.01	12.94 ± 0.83	44.50 ± 0.43
SCH2	50	5	HPβCD; 15	348.00 ± 39.10	0.623 ± 0.012	55.62 ± 0.42	0.17 ± 0.02	8.50 ± 0.08	35.06 ± 0.15
SCH3	50	10	HPβCD; 7.5	338.67 ± 35.23	0.449 ± 0.002	55.66 ± 0.46	0.34 ± 0.02	19.52 ± 0.35	39.11 ± 0.22
SCH4	50	10	HPβCD; 15	322.67 ± 37.81	0.427 ± 0.004	69.57 ± 0.58	0.13 ± 0.01	20.24 ± 0.96	41.72 ± 0.23
SCH5	50	15	HPβCD; 7.5	375.50 ± 14.85	0.228 ± 0.007	19.36 ± 0.32	0.24 ± 0.01	20.75 ± 0.35	42.84 ± 0.41
SCH6	50	15	HPβCD; 15	325.00 ± 46.78	0.224 ± 0.017	35.73 ± 0.19	0.13 ± 0.00	16.55 ± 0.33	30.47 ± 0.68
SCH7	70	5	HPβCD; 7.5	594.00 ± 28.28	0.746 ± 0.006	78.71 ± 0.21	0.35 ± 0.01	15.86 ± 0.16	39.90 ± 0.59
SCH8	70	5	HPβCD; 15	510.50 ± 10.61	0.756 ± 0.007	84.42 ± 0.37	0.25 ± 0.04	8.92 ± 0.09	28.85 ± 0.40
SCH9	70	10	HPβCD; 7.5	407.00 ± 21.92	0.518 ± 0.006	57.43 ± 0.29	0.25 ± 0.01	15.25 ± 0.09	36.32 ± 0.06
SCH10	70	10	HPβCD; 15	484.75 ± 33.87	0.550 ± 0.007	72.54 ± 0.45	0.15 ± 0.00	9.44 ± 0.57	32.13 ± 0.01
SCH11	70	15	HPβCD; 7.5	390.67 ± 34.27	0.314 ± 0.009	44.37 ± 0.24	0.22 ± 0.02	16.13 ± 0.47	43.00 ± 0.71
SCH12	70	15	HPβCD; 15	457.00 ± 49.81	0.338 ± 0.003	63.41 ± 0.37	0.16 ± 0.01	11.48 ± 0.58	14.75 ± 0.40

* TEOS and cryoprotectant concentrations are calculated as the percentage of the hydroalcoholic solution. Each formulation contained 0.5 mg/mL of terconazole and 0.1%*w*/*v* of chitosan HCl Abbreviations—TEOS: tetraethyl ortho silicate; MβCD: methyl-β-cyclodextrin; HPβCD: hydroxypropyl-β-cyclodextrin; PS: particle size; PDI: polydispersity index; DL: percentage drug loading; and Q_2_ and Q_6_: percentage of drug released after 2 and 6 h, respectively.

**Table 2:** Pharmacokinetic parameters of terconazole following the ocular administration of drug suspension and the selected formulation. SCM8-1 to the rabbits’ eyes.

Pharmacokinetic Parameter	Drug Suspension	SCM8-1
C_max_ (ng/mL)	752.2 ± 81.2	3449.3 ± 148.6
t_max_ (h) *	1	7
AUC (ng.h/mL)	2494.1 ± 274.0	49197.4 ± 5235.7
MRT (h)	4.9 ± 0.3	8.3 ± 0.2

* Values represent median.

## Data Availability

Data available within the article.
